# A Survey on the Use of Artificial Intelligence by Clinicians in Dentistry and Oral and Maxillofacial Surgery

**DOI:** 10.3390/medicina58081059

**Published:** 2022-08-05

**Authors:** Tim Eschert, Falk Schwendicke, Joachim Krois, Lauren Bohner, Shankeeth Vinayahalingam, Marcel Hanisch

**Affiliations:** 1 Department of Oral and Maxillofacial Surgery, Hospital University Muenster, 48149 Muenster, Germany; 2 Department of Oral Diagnostics and Digital Health and Health Services Research, Charité—Universitätsmedizin Berlin, 14197 Berlin, Germany; 3 Nijmegen Medical Centre, Department of Oral and Maxillofacial Surgery, Radboud University, P.O. Box 9101, 6500 HB Nijmegen, The Netherlands

**Keywords:** artificial intelligence, machine learning, qualitative research, clinicians survey, perception

## Abstract

*Background:* Applications of artificial intelligence (AI) in medicine and dentistry have been on the rise in recent years. In dental radiology, deep learning approaches have improved diagnostics, outperforming clinicians in accuracy and efficiency. This study aimed to provide information on clinicians’ knowledge and perceptions regarding AI. *Methods:* A 21-item questionnaire was used to study the views of dentistry professionals on AI use in clinical practice. *Results:* In total, 302 questionnaires were answered and assessed. Most of the respondents rated their knowledge of AI as average (37.1%), below average (22.2%) or very poor (23.2%). The participants were largely convinced that AI would improve and bring about uniformity in diagnostics (mean Likert ± standard deviation 3.7 ± 1.27). Among the most serious concerns were the responsibility for machine errors (3.7 ± 1.3), data security or privacy issues (3.5 ± 1.24) and the divestment of healthcare to large technology companies (3.5 ± 1.28). *Conclusions*: Within the limitations of this study, insights into the acceptance and use of AI in dentistry are revealed for the first time.

## 1. Introduction

Artificial intelligence (AI) in medicine and dentistry has drawn the attention of researchers in recent years because of its multiple applications [1,2]. AI is a decision-making and problem-solving model [3]. Convolutional neural networks (CNNs) learn structural patterns of a given dataset (input) and perform tasks autonomously, resulting in a data-based output [4]. In machine learning, CNNs mimic human neurons [5], creating a network organized in layers that transfers complex input of data (e.g., images, radiographs) into output data (e.g., diagnosis, planning) [4]. In the last two decades, deep learning has significantly improved machine learning, due to its deep CNN architecture that learns and performs complicated tasks without human assistance [3], making clinical applications, such as computer-aided diagnosis, possible [6]. In this context, the Food and Drug Administration and Conformité Européenne approved radiograph-analyzing software [7].

Functional applications of AI in dentistry include assisted treatment planning, computer-aided diagnosis based on medical images and predictive data analytics [2]. The relevant literature on dental medicine includes the evaluation of treatment decisions [8], the survival prediction of patients with oral cavity carcinoma [9] and the diagnosis of caries using radiographs [10]. In dental radiology, deep learning approaches have improved diagnostics by outperforming clinicians in accuracy and efficiency [11,12]. Furthermore, they permit decreasing the time spent on tasks and the number of cases of missed findings, and they prevent overtreatment [1,5,7].

Nevertheless, the debate over sensitive data, privacy security and ethical concerns remains present in the research, public, political and industrial sectors. The uncertainty of the responsibility for machine errors and the currently vague guidelines require policymakers and medical professionals to seek legal clarification [13].

The misperception of AI might result in unsubstantiated concerns. So far, most studies investigated the perception of AI for medical professionals or for medicine and dentistry students, but only a few focused on dental professionals, with all research having been conducted outside Europe [14,15,16,17,18,19,20,21,22,23]. Different educational standards in data and privacy security may result in a different legal clarification focus, depending on the location.

This study aimed to investigate dental clinicians’ knowledge and perceptions regarding AI. Giving professionals from the German district Westphalia-Lippe the opportunity to indicate the necessary steps for the introduction of AI in clinical settings is important in the context of coordinating a comprehensive educational program.

## 2. Materials and Methods

A 21-item questionnaire (Table A1) was used, adapted from the survey design by Scheetz et al. [21], who validated the survey through a literature review and consultation with medical specialists. A pilot test of the questionnaire was performed in advance. Disagreement on any questions was resolved by consensus (TE, SV, MH). This survey was approved by the Ethics Committee of the Westphalia-Lippe Medical Association, Westfälische-Wilhelms University Münster (decision no. 2021-616-f-S). This study was conducted in accordance with the code of ethics of the World Medical Association (Declaration of Helsinki).

### 2.1. Sample Size

Sample size estimation and power calculation were waived. This study was purely explorative and observational without concrete hypothesis testing.

### 2.2. Study Design

This prospective anonymous online survey was conducted between December 2021 and March 2022. Invitations were sent to a random sample of 1500 dentists, specialist dentists and oral and craniomaxillofacial surgeons randomly chosen from the membership list of the Dental Association of Westfalen-Lippe, Germany. Participation was voluntary, and no incentives were provided. Informed consent was signed online before starting the survey. The questionnaire included demographic data and questions concerning AI-related knowledge, potential impact, expectations, advantages and concerns. The data were collected and managed using Q-Set.

### 2.3. Statistical Analysis

The descriptive statistical analysis was performed on SPSS Version 28.0 (IBM, Armonk, NY, USA). A two-sided chi-square test or two-sided Fischer’s test was used to calculate group differences. For an asymptomatic approximation, the Monte Carlo method was used when computational time was invalid. A *p*-value of <0.05 was set as the level of significance.

## 3. Results

**Figure 1:**
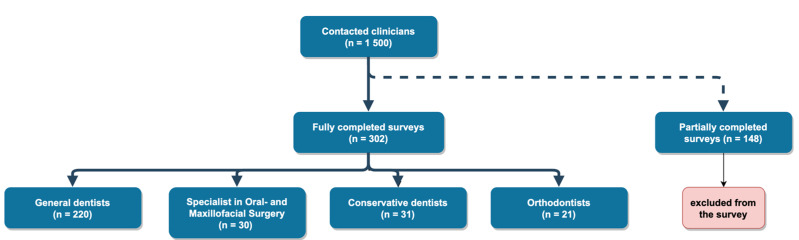
Flow chart describing the compilation of the survey participants.

From the 1500 invited clinicians, 450 questionnaires were answered, 148 of which were excluded as they were incomplete. A total of 302 questionnaires were assessed. As for the professions, 220 participants were general dentists, 30 were specialists in oral and maxillofacial surgery, 21 were orthodontists and 31 were specialists in conservative dentistry, including endodontics and periodontology (Figure 1). The majority of the respondents (43.4%) were in the age range of 46 to 60.

More than half of the participants practiced in an urban (36.1%) or somewhat urban (24.8%) environment (*p* = 0.054). Oral and maxillofacial surgeons were predominantly based in a metropolitan setting (somewhat urban/urban: 73.3%; *p* = 0.048).

### 3.1. Status of Knowledge and Use of AI in the Daily Workflow

**Figure 2:**
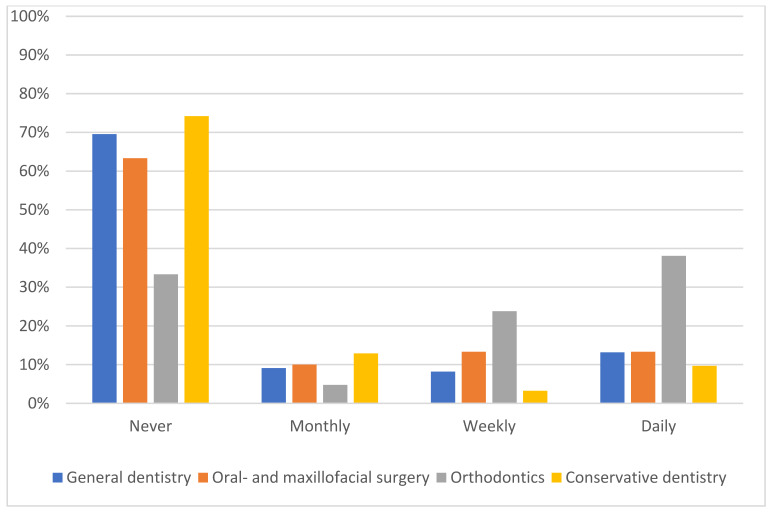
Self-reported frequency of the use of AI in clinical practice.

Most of the respondents rated their knowledge of AI as average (37.1%), below average (22.2%) or very poor (23.2%). Only 6.3% reported having excellent AI knowledge. Orthodontists and oral and maxillofacial surgeons (38.1% and 36.7% of total respondents, respectively, reported having above average/excellent AI knowledge) reported having better knowledge of AI (*p* = 0.003). This imbalance also applies to the frequency of use. Orthodontists are almost three times more likely to use AI on a daily or weekly basis than their colleagues in other professions (61.9% of orthodontists; 21.0% summarizing GD, CD, CMF-Surgeon; *p* = 0.018) (Figure 2). Most of the participants (66.9%) stated that they never use AI in daily practice. Clinicians reported having better knowledge when the use of AI is frequent. A total of 47.7% of the dentists using AI daily rated their knowledge as above average or better. In comparison, only 9.8% of the dentists using AI infrequently rated their knowledge as above average or better (*p* < 0.001). An open-ended question was used to determine which AI-based applications participants use in daily clinical practice. While most answers indicate that the respondents do not use AI at all, the most mentioned application focused on radiology (*n* = 21). For most parts, the exact area of application remained unclear, and some participants specified using software like “dentalXrai”. The second and third most relevant use included intraoral-scanning (*n* = 20) and aligner treatment or planning (*n* = 10) (Table 1).

### 3.2. Predicted Impact of AI

**Figure 3:**
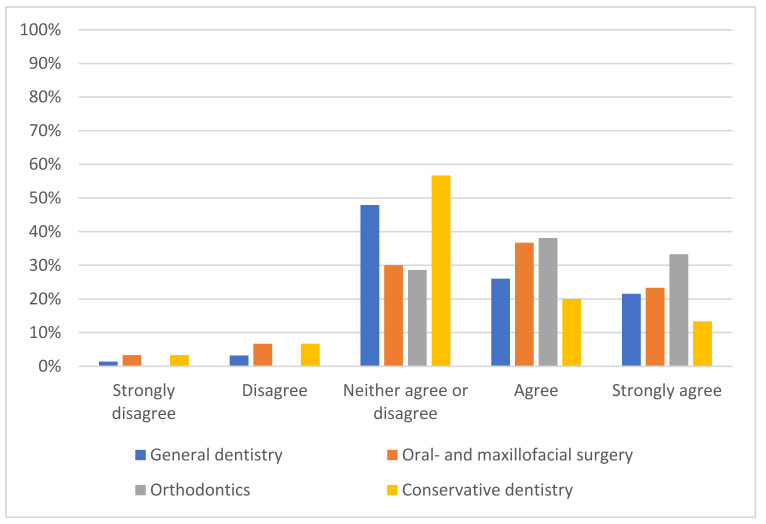
Approval rating of the statement “The introduction of AI will lead to improvement in my profession”.

The majority of the participants expected AI to impact their profession within 5 years (43%) or 5–10 years (36.1%), with almost half of them (49%) predicting a positive impact (Figure 3). Almost half of the respondents thought that AI would reduce the workforce in healthcare (44.7%), while almost the same portion predicted no changes in the number of healthcare workers (43.0%).

### 3.3. Tolerance Level of Faulty AI Performance

The majority of the participants desired AI performance to be superior to an average clinician (35.1%) or superior to the best-performing clinician (34.8%) in disease-screening support. Rural-based practitioners had lower expectations regarding AI performance in disease-screening support (equivalent to an average performing clinician or lower: 42.9%) than clinicians practicing in a metropolitan area (equivalent to or lower than an average performing clinician: 24.8%; *p* = 0.034). The expectations for acceptable performance standards in clinical decision support were higher (superior to an average performing clinician: 34.1%; better than an average performing clinician: 41.4%). There were no other statistically significant features (profession, age or experience with AI) associated with the responses.

### 3.4. Perceived Advantages of AI

The participants were largely convinced that AI would improve diagnostics by bringing about uniformity (mean Likert ± standard deviation 3.7 ± 1.27). However, the clinicians did not expect AI to influence referrals to specialists (3.0 ± 1.17) and rated the impact on cost-effectiveness as not relevant (2.9 ± 1.17) (Table 2).

### 3.5. Perceived Concerns over AI

While the positive impact of AI on the diagnostic process was rated as the most important influence of AI on dentistry, the responsibility for machine errors (mean Likert ± standard deviation 3.7 ± 1.3), data security or privacy issues (3.5 ± 1.24) and the divestment of healthcare to large technology companies (3.5 ± 1.28) were among the most serious concerns (Table 3).

### 3.6. Perceived Preparation of the Workplace for the Introduction of AI

Clinicians that reported never using AI are significantly more convinced that their workplace is not adequately prepared for the introduction of AI (67.3%, total of clinicians never using AI) than clinicians using AI daily (43.2%). However, 81.1% of the participants could imagine implementing a workflow that is based on AI for diagnostics support. There were no other statistically significant features (profession, age or experience with AI) associated with the responses.

## 4. Discussion

The perception of dentists and oral and maxillofacial surgeons regarding AI will play a major role in its successful implementation in healthcare. This study aimed to display dentistry professionals’ attitudes, knowledge, prospects and prognoses regarding the use of AI models in the clinical context. Many studies have investigated the perception of dental or medical students towards AI, but no study of dental and maxillofacial healthcare professionals in Europe had been conducted so far [14,16,17,19,20,21,22,23,24].

This study showed that the frequent use of AI in daily practice is a key factor for adequate knowledge in this area. The majority of the respondents that rated their knowledge as excellent (63.2%) use AI daily or weekly (*p <* 0.001). However, a lack of AI knowledge results in a negative attitude towards this technology [23]. One of the reasons for the frequent use of AI in the dental workflow may be the spread of aligner therapy that includes AI-guided treatment planning [25] or intraoral scanning [26].

Clinicians favoured the following major advantages of AI models: improved and uniform diagnosis combined with individual and evidence-based treatment. Other studies showed similar results [15,16,22]. In this context, radiologists rated the reduced time required for monotonous tasks as an important AI-derived improvement [22]. Dentists are requested to interpret a variety of radiographs (e.g., cone beam computed tomography, panoramic radiograph, bitewing, lateral cephalogram, etc.) [27]. Depending on the clinicians’ experience with different X-ray techniques, diagnoses vary widely and often deviate from the actual diagnostic findings [27]. The time-consuming diagnostic process can be efficiently and accurately performed by AI [3,10,28], allowing clinicians to dedicate more time to patient care. A significant increase in patient satisfaction has been shown when the clinician spends more time with the patient [29,30].

Concerns regarding the responsibility for AI-induced errors, the divestment of healthcare to large data and technology companies, as well as privacy and data security matters, were crucial for the participants and confirm the findings of surveys with other medical professions [22]. To increase trust in this technology, additional education on that topic is needed [5]. This is supported by the participants’ most common proposal, providing information relevant to AI use in dental seminars, congresses and professional meetings. Similar proposals have been documented elsewhere [22,23].

Despite several serious concerns, clinicians in various surveys agree on the positive impact of AI on their profession [15,17,18,20,23,24]. In a study with medical students from Germany, 83.7% expected AI-derived improvement in medicine in general [15], and students from nine Turkish dental schools showed similar results, with 85.7% agreeing that AI will lead to major advances in the dental sector [24]. This shows that the results of the present study are not only comparable to other surveys of dental healthcare professionals but also applicable to other medical areas. The impact of the supporting role of AI on all medical and dental professions will be evident in multidisciplinary areas and the daily clinical routine.

AI will continue to assist the clinician in the decision-making process by connecting information that otherwise would be difficult to collect and compare, especially over time [3]. Besides the support in diagnostics, it will boost efficiency and accuracy [3,10,28]. Devito et al. [31] achieved an improvement in the diagnosis of proximal caries by 39.4% using an artificial neural network when compared with 25 examiners’ diagnoses [31]. Cantu et al. compared dentists against a neural network, wherein the latter showed a significantly higher accuracy in caries detection, in particular for early caries lesions [10].

Along with those aspects, it is crucial to emphasize the supporting role of AI. In the near future, the decision process will not be conducted by AI systems alone, and the possibility of replacing clinicians is unrealistic [32]. The clinicians in this survey showed no significant difference regarding their opinion on AI’s impact on the required workforce. An investigation concerning specialists’ fears of being replaced by AI showed that it was rated as unlikely for most of the participants to be replaced by an AI (83%) [15]

Pauwels et al. documented decreased skepticism after a lecture about AI [23]. Additional education in this area may reduce negative attitudes towards AI and help make it an accepted tool in practitioners’ daily routines. Promoting AI in education, the inclusion of all stakeholders in the development process and ensuring a legal and ethical basis will be key elements for the success of AI in dentistry and medicine.

Furthermore, this study serves as a basis for future quantitative studies on this topic. More specifically, prospective studies may focus on the quality and effect of the implemented educational programs and guidelines to demonstrate the impact of AI models on dentistry practice.

### Limitations

The sample size of this study (302 participants with memberships in the Dental Association of Westfalen-Lippe, Germany) is not representative of all regions of Germany. Due to voluntary participation, response bias cannot be excluded.

## Figures and Tables

**Table 1:** Applications of artificial intelligence (AI) mentioned in an open-ended question.

Application	Appearance (*n*)	%
No use of AI in clinical practice	31	28.7%
Radiology and diagnosis with radiographs	21	19.4%
Intraoral scanning	20	18.6%
Aligner treatment	10	9.3%
CAD/CAM *	9	8.3%
Implantology	9	8.3%
Treatment planning	8	7.4%
Total	108	100%

* Computer-aided design (CAD)/Computer-aided manufacturing (CAM).

**Table 2:** The advantages of AI in dentistry according to 302 dentists rated on a 5-point Likert scale. 1: low relevance; 5: high relevance.

Statements	Mean (SD)
Improved diagnostics	3.7 (1.3)
Uniformity in diagnostics	3.6 (1.1)
More individual and evidence-based health care	3.4 (1.1)
Reduced time on monotonous tasks	3.2 (1.3)
Improvement in disease prediction	3.2 (1.1)
Improved access to disease screening	3.1 (1.2)
More targeted referrals to specialists	3.0 (1.2)
More cost-efficient health care	2.9 (1.2)

**Table 3:** The concerns over the use of AI in dentistry according to 302 as rated on a 5-point Likert scale. 1: a little concerning; 5: highly concerning.

Statements	Mean (SD)
Concerns over liability and responsibility for machine errors	3.7 (1.3)
Concerns over data security and privacy issues	3.5 (1.2)
Concerns over the divestment of healthcare to technology companies	3.5 (1.3)
Lack of trust in the diagnostic capability of AI	3.1 (1.0)
Concerns over a reduced need for specialists	3.0 (1.2)
Challenge for the patient–doctor relationship	2.8 (1.2)
Concerns regarding the comparison between clinicians and AI	2.8 (1.2)
Negative impact on the workforce	2.7 (1.2)

## Data Availability

Data are available by the authors.

## Appendix A

**Table A1:** Questionnaire.

1.	How old are you?	18–25		26–45		46–60		
2.	What is your profession?	General dentistry			Periodontology			
		Oral- or maxillofacial surgery			Orthodontics			
		Endodontics			Pedodontics			
		Other:			[text]			
3.	Since when do work in your profession?	In training		<5 years		5–10 years		
		20–30 years		<30 years				
4.	Work environment	Rural	Urban		Somewhat rural		Somewhat urban	
5.	How often do you use artificial intelligence in your daily work?	Never		Monthly		Weekly		
6.	What are the areas of application you use AI for?	[text]						
7.	In comparison to your colleagues in your profession how would you rate your knowledge in the topic of AI and it’s application possibilities in your profession?	Excellent			Above average			
		Average			Below average			
		Very poor						
8.	How long will it take in your opinion until AI has a noticeable impact on your profession?	<1 year			1–5 years			
		5–10 years			>10 years			
		never						
9.	To what extent do you expect AI to impact the workforce needed in your profession in this decade?	To a great extent		Somewhat		Very little		
10.	To what extent do you expect AI to impact the workforce needed in your profession in the next decade?	To a great extent		Somewhat		Very little		
11.	How will AI impact the workforce needed?	Increase		Decrease		None		
12.	Is your profession adequately equipped for the application of AI?	Yes		No		Unsure		
13.	What measures should be taken to prepare your profession for the application of AI?	[text]						
14.	What degree of error tolerance is acceptable for an AI based model that is used for disease screening by non-specialized health care workers?	Equivalent to the worst performing			Equivalent to the average performing			
		Superior to the average performing			Equivalent to the best performing			
		Superior to the best performing						
15.	What degree of error tolerance is acceptable for an AI based model that is used for support of a diagnostic decision by specialists?	Equivalent to the worst performing			Equivalent to the average performing			
		Superior to the average performing			Equivalent to the best performing			
		Superior to the best performing						
16.	Can you imagine implementing the following workflow in your clinical life: Radiographs of a patient are diagnosed by an AI. A specialist evaluates the radiographs and the AI’s findings.	Yes		No		Unsure		
17.	Which of the following advantages regarding the application of AI in clinical life are most important? Evaluate from 1–5 (1 = least significant, 5 = most significant)	Better access to disease-screening			More targeted referrals			
		More cost-efficient healthcare			Better diagnostics			
		Less time-consuming monotonous tasks			More consistent diagnostics			
		More individual and evidence-based treatment			Better prediction of the course of disease			
18.	Which of the following aspect are the most concerning regarding the application of AI in clincal life? Evaluate from 1–5 (1 = most concerning, 5 = least concerning)	Concerns regarding the outsouring of the steps of procedure to large data and technology companies			Privacy and data security concerns			
		Concerns over accountability and responsibility in case of machine errors			Lack of trust in diagnostic capability of the AI			
		Reduced demand for specialist groups			Challenge for the patient–doctor relationship			
		Concerns regarding the benchmarking between clinicians and AI			Consequences for the workforce			
19.	Which of the following professions will profit most of the introduction of AI?	Endodontics			Orthodontics			
		Pedodontics			Conservative Dentistry			
		Oral- and maxillofacial surgery			Periodontics			
		Prosthodontics			Other: [text]			
20.	To what extent do you agree with the following statement:„The introduction of AI will lead to improvement in my profession.”	Strongly agree			Agree			
		Neither agree or disagree			Disagree			
		Strongly disagree						
21.	To what extent do you agree with the following statement:„The introduction of AI will reduce iatrogenic errors in my profession.”	Strongly agree			Agree			
		Neither agree or disagree			Disagree			
		Strongly disagree						
